# Patch testing in drug cutaneous adverse reactions: value of the extemporaneous preparations

**DOI:** 10.1186/2045-7022-4-S3-P105

**Published:** 2014-07-18

**Authors:** Haudrey Assier, Gwendoline Gener, Laurence Valeyrie-Allanore, Pierre Wolkenstein, Olivier Chosidow

**Affiliations:** 1 Department of Dermatology, France; 2 Henri Mondor Hospital, Department of Dermatology, France

## Background

Patch testing (PT) is a part of tools used for the etiological diagnosis of a cutaneous adverse reaction (CADR). Positivity of PT is observed in above 30 to 50 % of explored cases. Recommended procedure is based on dilution in petroleum or in water at 30 % for the finished product or at 10 % for the active ingredient. Chemotechnique laboratory supplies, in the form of ready for use syringes, around 30 active ingredients. Other medicines must be thus prepared by a hospital pharmacy, according to the strict protocol. Thus, preparations are not available in all the test centers and some patients can not be explored. We led a forward-looking study comparing the extemporaneous tests, easily practicable, compared with the recommended procedures. Patients and methods The programmed tests were performed at the same time with Chemotechnique products or pharmaceutical preparations and with tablets brought by the patients, crushed manually with a pestle in order to transform in powder and then mixed in petroleum. The used chambers were Q or IQ chambers ( Chemotechnique ) and the readings realized at 48 and 96 hours.

## Results

70 patients (25 men/ 45 women) were included. Seven were tested with 2 different molecules, thus 77 double tests were performed. Tested substances included usual CADR inducers such as allopurinol (n=9), amoxicillin (n=25), tetrazepam (n=7), pristinamycin (n=10) and 16 others molecules. CADR tested included maculopapular rash (MPR; n=27), Drug Reaction with Eosinophilia and Systemic Symptoms (DRESS; n=9), Acute Generalized Exanthematous Pustulosis (AGEP; n=12), Fixed Drug Reaction (FDE; n=5), Linear IgA Dermatosis (n=2), Stevens-Johnson Syndrome/Toxic Epidermal Necrolysis (n=15), erytroderma (n=3) and eczematous rash (n=2). All the tests were coherent: 20 positive tests and 57 negative with the 2 techniques. As usually observed in literature with recommended patch testing, the positive tests were observed preferentially with some ingredients (amoxicillin, tetrazepam, pristinamycin) and conversely there was no positive tests with allopurinol, ibuprofen and piroxicam. As well, patch testing was more frequently positive with some kinds of CADR (AGEP and MPR), confirming reliability of the tests.

## Conclusion

For the patients that could not consult easily in centers having access to the pharmaceutical preparations but nevertheless expert in CADR, the extemporaneous preparations constitute a possible alternative.

